# Alcohol consumption is inversely associated with stage 3 chronic kidney disease in middle-aged Taiwanese men

**DOI:** 10.1186/1471-2369-14-254

**Published:** 2013-11-17

**Authors:** Yueh-Han Hsu, Hsiang-Chu Pai, Yao-Mao Chang, Wen-Hsin Liu, Chih-Cheng Hsu

**Affiliations:** 1 Department of Health Services Administration, China Medical University, Taichung City 404, Taiwan; 2 Department of Internal Medicine, Division of Nephrology, Ditmanson Medical Foundation Chia-Yi Christian Hospital, Chia-Yi City 600, Taiwan; 3 Department of Nursing, Min-Hwei Junior College of Health Care Management, Tainan City 736, Taiwan; 4 Center for Liberal Arts, Taipei Medical University, Taipei 110, Taiwan; 5 Division of Family Medicine, Ditmanson Medical Foundation Chia-Yi Christian Hospital, Chia-Yi City 600, Taiwan; 6 Institute of Population Health Sciences, National Health Research Institutes, Zhunan, Miaoli County 35053, Taiwan; 7 Department of Health Services Administration, China Medical University and Hospital, Taichung City 404, Taiwan

**Keywords:** Alcohol consumption, Chronic kidney disease, Gender, Proteinuria, Taiwan

## Abstract

**Background:**

Chronic kidney disease (CKD) is a major global public health burden, but there is limited understanding of the relationship of alcohol consumption with CKD.

**Methods:**

In this cross-sectional multivariable study, all participants of a health check-up program in Ditmanson Medical Foundation Chia-Yi Christian Hospital in Taiwan from 2003 to 2009 (15 353 women and 11 900 men) were included for analysis. Estimated glomerular filtration rate was used to define CKD stage and history of alcohol consumption was obtained by self-reporting. Multivariable logistic regression analyses of gender-specific association of alcohol drinking with stage 3 CKD were conducted. A trend tests was conducted to check the dose–response relationship of alcohol consumption with renal disease. A sensitivity test was conducted to rule out the likelihood of reverse causality.

**Results:**

The prevalence of stage 3 CKD was lower in drinkers than non-drinkers (*p <* 0.001) and the percentage of drinkers with stage 3 CKD was less than that of non-drinkers. Multivariable analysis indicated that alcohol consumption was negatively associated with the presence of stage 3 CKD in men (adjusted odds ratio [aOR] for occasional drinking: 0.68, 95% CI: 0.59 ~ 0.78, *p <* 0.001; aOR for frequent drinking: 0.47, 95% CI: 0.35 ~ 0.63, *p <* 0.001). Advanced age, hypertension, anemia, BMI of at least 24, hyperuricemia, and proteinuria were also associated with stage 3 CKD in men. Trend tests indicated lower odds of having stage 3 CKD with increased alcohol consumption in both genders. Subgroup analyses and sensitivity tests also indicated the reverse association between alcohol consumption and stage 3 CKD in men regardless of age, diabetes status, and other risky behaviors.

**Conclusions:**

Alcohol consumption was inversely associated with stage 3 CKD in Taiwanese men. However, considering the potential of other health damage with alcohol consumption, the current results should be interpreted cautiously.

## Background

Chronic kidney disease (CKD) and end-stage renal disease (ESRD) are among the most significant non-communicable diseases [1-3]. Taiwan has the highest prevalence of ESRD world-wide [4], bringing a significant disease burden in the society. Early detection of factors that increase or decrease the risk of CKD may help to reduce this disease burden.

Many recent studies have focused on patients with stage 3 CKD because such patients have increased risk of renal failure and cardiovascular events [5-7]. A longitudinal follow-up study indicated that death was more common than initiation of dialysis in patients diagnosed with stage 2 to 4 CKD [8]. In addition, modest reductions in the renal function of patients with stage 3 CKD are associated with reduced survival [9]. Among Taiwanese patients with stage 3 to 5 CKD (6.9% of the Taiwanese adult population), 6.5% of patients had stage 3 disease and 0.4% of patients had stage 4 to 5 disease, but patients with stage 3 disease had much lower awareness of their condition (8% *vs.* 25 ~ 71.4%) [3].

Alcohol is one of most frequently used psychotropic substances in the world. Long-term excessive alcohol drinking is widely accepted as a risk factor for cardiovascular diseases including hypertension and cerebro-vascular events [10]. However, the results of previous research on the association of alcohol drinking with CKD and ESRD have been conflicting. Consumption of more than two drinks per day was associated with increased risk of ESRD in an American population [11]. Daily drinking of 4 or more servings, especially when combined with smoking, was independently associated with increased CKD risk in another American population (defined as estimated glomerular filtration rate [eGFR] < 60 mL/min/1.73 m^2^) [12]. Further, alcohol consumption of 30 g or more daily was associated with an increased risk of albuminuria in an Australian population [13]. However, Savdie et al. reported that consumption of three or more drinks per day was associated with a decreased level of serum creatinine (SCr), but that consumption of two or fewer drinks daily was associated with a higher level of SCr [14]. Several recent large prospective series also reported an inverse association of alcohol consumption and CKD (defined similarly), but this association was only present in those who drank moderately [13,15,16].

A recent study of a Chinese population indicated a significant inverse relationship between alcohol consumption and risk of ESRD, and that this risk was much lower for heavy drinkers (more than 21 drinks per week) [17]. However, to our best knowledge, there is limited research on the relationship of alcohol drinking with stage 3 CKD. Furthermore, most studies of the relationship of drinking with renal disease have not fully controlled for potential confounding covariates, such as anemia and hyperuricemia, raising a concern about the validity of this relationship.

Betel nut (BN) chewing is a common practice in Taiwan [18]. The prevalence of BN chewing in Taiwanese men was reported to be over 28% [19]. Most Taiwanese people who chew BN also consume alcohol and cigarettes [20]. BN chewing was reported to be associated with CKD and proteinuria in Taiwanese population [20,21].

The aim of the present research was to investigate the association between alcohol consumption and stage 3 CKD in males and females from Taiwan by using a large dataset (n = 27 253) with statistical adjustment for factors including anemia, hyperuricemia, smoking, and BN chewing.

## Methods

### Study subjects

Detailed description and definitions of variables in the dataset were described previously [20]. Briefly, from 2003 to 2009, 34 372 people who attended a national health insurance sponsored health check-up program in Ditmanson Medical Foundation Chia-Yi Christian Hospital, were selected as study subjects. Among the 1481 participants who attended the program more than one time, only the first check-up records were included. Participants were excluded if their records were incomplete (n = 5409) or if they had advanced renal dysfunction (eGFR < 30 mL/min/1.73 m^2^, n = 246) because such patients may rapidly progress to ESRD. The project was approved by the Institutional Review Board of Ditmanson Medical Foundation Chia-Yi Christian Hospital.

### Clinical and demographic variables

All participants received lab tests and completed questionnaires on past personal medical history and health-related behaviors with the assistance of trained volunteers. The questions asking about drinking status read “in the past 6 months, how often did you drink: did not drink, drank occasionally, or drank frequently?” Based on this question, the participants were classified into non-drinkers, occasional drinkers, or frequent drinkers, accordingly. Questions and criteria to define cigarette smokers and BN chewers were similar. The demographic and clinical survey (measured with standard automated technology after 8 hours of overnight fasting, see below) recorded age, sex, cigarette smoking, alcohol consumption, BN chewing, personal medical history (CKD, diabetes mellitus [DM], hypertension [HTN], hyperlipidemia, hyperuricemia, liver dysfunction, and anemia), systolic blood pressure (SBP), diastolic blood pressure (DBP), SCr, total cholesterol (TC), triglycerides (TG), uric acid (UA), fasting blood glucose (FBG), alanine aminotransferase (ALT), hemoglobin (Hb), white blood cell (WBC) count, and urinalysis (including biochemical and microscopic examination of sediment). Height and weight were used to calculate body mass index (BMI) and the Modification of Diet in Renal Disease (MDRD) formula was used to calculate eGFR [22].

### Definitions

CKD was defined by the presence of an eGFR less than 60 but at least 30 mL/min/1.73 m^2^. Proteinuria was defined as a grade of 1+ to 4+ in a morning dipstick test. HTN was defined as a history of HTN as determined by a healthcare professional or a blood pressure higher than 140/90 mmHg, regardless of medication usage. DM was defined as fasting plasma glucose level of at least 126 mg/dL or a history of DM as determined by a healthcare professional, regardless of medication usage. Hyperlipidemia was defined as a serum TC level above 200 mg/dL, a TG level of at least 200/dL, or history of this condition as determined by a healthcare professional, with or without medication. Participants were defined as non-drinkers if they did not consume alcohol in the previous 6 months, as occasional drinkers if they consumed alcohol occasionally or socially, and as frequent drinkers if they drank frequently or regularly, regardless of the amount they drank. Similar criteria were used to define smokers and BN chewers. The questionnaire inquiring health behaviors was filled out by the participants with the assistance of trained volunteers. Liver dysfunction was defined as an ALT level greater than 44 IU/L. Anemia was defined as an Hb level less than 13 g/dL in men and less than 12 g/dL in women who were not pregnant. Hyperuricemia was defined as a serum UA level greater than 7.0 mg/dL [23].

### Laboratory measurements

ALT, TC, TG, UA, FBG and SCr were measured by an automated analyzer (Hitachi 7170, Hitachi High Technologies Co, Tokyo, Japan). The test agent for ALT was manufactured by Roche Diagnostics GmbH (Germany) and test agents for all other lab tests were manufactured by Wako Pure Chemical Industries, Ltd. (Japan). Hb and WBC counts were measured by an automated analyzer (Sysmex XE-2100/5000, Sysmex Co., Japan). Dipstick urinalysis was performed by an automated chemical analyzer (URISYS 2400, Roche Diagnostics, Germany).

### Statistical analysis

Men and women had different drinking behaviors, so data of the two genders were analyzed separately. Baseline characteristics are expressed as means ± SD for continuous variables, and as counts and proportions for categorical variables. Student’s *t-*test and the chi-square test were used to compare continuous and categorical variables, respectively. Analysis of covariance was conducted to compare the age-standardised trends of serum creatinine and eGFR in both genders with regards to the levels of alcohol drinking. Traditional risk factors and potential confounding factors for CKD were included as covariates in a three-step multivariable logistic regression analysis to assess the independent association of alcohol drinking with CKD. The adjusted covariates in this study were age, smoking, BN chewing, BMI, HTN, DM, hyperlipidemia, hyperuricemia, liver dysfunction, anemia, and proteinuria. Results are expressed as odds ratio (OR) and 95% confidence intervals (CIs). The Cochran-Armitage test was used to assess the trend of alcohol consumption and CKD by treating the 3 different drinking classes (none, occasional, frequent) as continuous variables. Individuals were further stratified by age, diabetes status, and lifestyle status for subgroup multivariable analyses and sensitivity tests to control for confounding effects and likelihood of reverse causality. A two-sided *p*-value less than 0.05 was considered statistically significant. All calculations were performed with SPSS for Windows version 18 (SPSS Inc., Chicago, IL, USA).

## Results

**Figure 1:**
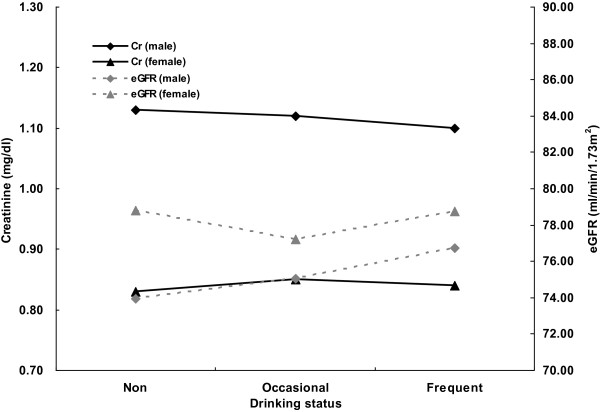
Age-adjusted means of serum creatinine (Cr) and estimated glomerular filtration rate (eGFR) in men and women in different alcohol drinking status.

We included 27 253 participants (15 353 women and 11 900 men, mean age: 57.90 ± 11.82 years) in the analysis. A total of 4 273 participants had stage 3 CKD, and 4 585 participants (16.8%) were self-reported drinkers of alcohol. A total of 84.7% of all drinkers were male, 32.6% of males were drinkers, and 4.6% of females were drinkers. Univariate analysis indicated that the prevalence of CKD was significantly lower in drinkers than in non-drinkers (10.6% *vs.* 16.7%, *p <* 0.001) and that this relationship held for females alone (8.7% *vs.* 14.7%, *p* < 0.001) and males alone (10.9% *vs.* 20.5%, *p* < 0.001). After age standardization, males who were frequent drinkers had better renal function than those who were occasional drinkers and non-drinkers. The trends in females were not present, which we believe might be related with small numbers of female drinkers (Figure 1).

Analysis of the clinical and demographic characteristics for enrolled participants (Tables 1 and 2) indicated that both male and female drinkers tended to be younger, have lower prevalence of CKD, HTN, and DM, lower levels of SCr, FBG, and SBP, higher levels of eGFR, and higher prevalence of smoking and BN chewing. The male drinkers tended to have lower prevalence of anemia and proteinuria, higher prevalence of hyperuricemia and liver dysfunction, and higher levels of BMI, UA, ALT, Hb, and WBC count.

**Table 1:** Demographic and clinical characteristics of men with different self-reported drinking histories

	**Overall**	**Non-drinkers**	**Occasional drinkers**	**Frequent drinkers**	***p***
**Variable**^**†**^	**(n = 11900)**	**(n = 8015)**	**(n = 3246)**	**(n = 639)**	
Age (years)	58.82 ± 12.04	60.87 ± 12.14	54.55 ± 10.66	54.85 ± 10.54	<0.001
Stage 3 CKD, n (%)	2064(17.3)	1640(20.5)	361(11.1)	63(9.9)	<0.001
eGFR (mL/min/1.73 m^2^)	74.41 ± 16.17	72.85 ± 16.24	77.38 ± 15.41	78.90 ± 16.07	<0.001
Creatinine (mg/dL)	1.13 ± 0.22	1.14 ± 0.23	1.10 ± 0.19	1.08 ± 0.19	<0.001
Hypertension, n (%)	5372(45.1)	3703(46.2)	1355(41.7)	314(49.1)	<0.001
Systolic BP (mmHg)	133.01 ± 19.56	133.19 ± 19.78	132.08 ± 19.02	135.52 ± 19.25	<0.001
Diastolic BP (mmHg)	79.26 ± 12.25	78.55 ± 12.13	80.25 ± 12.34	83.09 ± 12.37	<0.001
Hyperlipidemia, n (%)	432(3.6)	287(3.6)	124(3.8)	21(3.3)	0.739
Cholesterol (mg/dL)	206.68 ± 41.30	205.62 ± 40.15	209.54 ± 43.17	205.49 ± 45.06	<0.001
Triglyceride (mg/dL)	156.70 ± 166.51	143.66 ± 115.73	173.54 ± 228.20	234.76 ± 272.61	<0.001
Hyperuricemia, n (%)	4629(38.9)	2997(37.4)	1334(41.1)	298(46.6)	<0.001
Uric acid (mg/dL)	6.75 ± 1.62	6.69 ± 1.64	6.83 ± 1.52	7.02 ± 1.72	<0.001
Diabetes, n (%)	1767(14.8)	1233(15.4)	415(12.8)	119(18.6)	<0.001
Fasting blood glucose (mg/dL)	107.03 ± 40.24	106.86 ± 39.70	106.60 ± 40.65	111.37 ± 44.42	0.019
Liver dysfunction, n (%)	2480(20.8)	1581(19.7)	732(22.6)	167(26.1)	<0.001
ALT (IU/L)	37.14 ± 42.86	36.28 ± 44.58	38.32 ± 37.89	41.97 ± 44.22	0.001
Anemia, n (%)	719(6.0)	582(7.3)	109(3.4)	28(4.4)	<0.001
Hemoglobin (g/dL)	15.19 ± 1.44	15.06 ± 1.45	15.43 ± 1.32	15.55 ± 1.54	<0.001
WBC count (10^3^/μL)	6.51 ± 1.99	6.47 ± 1.95	6.57 ± 1.82	6.79 ± 3.01	<0.001
Proteinuria, n (%)	1310(11.0)	910(11.4)	308(9.5)	92(14.4)	<0.001
BMI (kg/m^2^)	25.11 ± 3.47	24.94 ± 3.48	25.50 ± 3.34	25.29 ± 3.76	<0.001
Smoking, n (%)	3024(25.4)	1425(17.8)	1192(36.7)	407(63.7)	<0.001
Betel nut chewing, n (%)	1538(12.9)	484(6.0)	760(23.4)	294(46.0)	<0.001

†In this table and Table 2, results are expressed as n (%) or mean ± SD.

**Table 2:** Demographic and clinical characteristics of women with different self-reported drinking histories

	**Over all**	**Non-drinkers**	**Occasional drinkers**	**Frequent drinkers**	***p***
**Variable**	**(n = 15353)**	**(n = 14653)**	**(n = 654)**	**(n = 46)**	
Age (years)	57.18 ± 11.59	57.49 ± 11.62	50.80 ± 8.77	48.74 ± 8.84	<0.001
CKD, n (%)	2209(14.4)	2148(14.7)	58(8.9)	3(6.5)	<0.001
eGFR (mL/min/1.73 m^2^)	78.74 ± 17.45	78.58 ± 17.49	81.78 ± 16.28	84.78 ± 16.51	<0.001
Creatinine (mg/dL)	0.83 ± 0.17	0.83 ± 0.17	0.82 ± 0.14	0.80 ± 0.13	0.005
Hypertension, n (%)	6187(40.3)	5998(40.9)	171(26.1)	18(39.1)	<0.001
Systolic BP (mmHg)	130.40 ± 21.18	130.69 ± 21.23	124.05 ± 19.02	127.70 ± 19.28	<0.001
Diastolic BP (mmHg)	75.14 ± 11.83	75.14 ± 11.81	74.65 ± 12.11	80.39 ± 13.29	0.006
Hyperlipidemia, n (%)	544(3.5)	524(3.6)	19(2.9)	1(2.2)	0.584
Cholesterol (mg/dL)	213.88 ± 41.00	214.06 ± 41.06	210.06 ± 39.42	211.37 ± 43.25	0.046
Triglyceride (mg/dL)	125.78 ± 96.20	126.17 ± 96.86	114.20 ± 73.61	167.43 ± 141.03	<0.001
Hyperuricemia, n (%)	1912(12.5)	1829(12.5)	78(11.9)	5(10.9)	0.868
Uric acid (mg/dL)	5.44 ± 1.44	5.44 ± 1.45	5.44 ± 1.37	5.67 ± 1.13	0.538
Diabetes, n (%)	1801(11.7)	1758(12.0)	42(6.4)	1(2.2)	<0.001
Fasting blood glucose (mg/dL)	102.69 ± 35.94	102.94 ± 36.36	97.50 ± 25.49	96.15 ± 16.30	<0.001
Liver dysfunction, n (%)	1894(12.3)	1797(12.3)	88(13.5)	9(19.6)	0.217
ALT (IU/L)	28.92 ± 38.35	28.93 ± 38.77	28.50 ± 28.79	28.93 ± 18.77	0.960
Anemia, n (%)	1711(11.1)	1632(4.3)	73(11.2)	6(13.0)	0.919
Hemoglobin (g/dL)	13.36 ± 1.32	13.36 ± 1.32	13.36 ± 1.36	13.47 ± 1.41	0.847
WBC counts (10^3^/μL)	6.00 ± 1.73	6.00 ± 1.73	5.96 ± 1.66	6.31 ± 1.92	0.390
Proteinuria, n (%)	1326(8.6)	1278(8.7)	43(6.6)	5(10.9)	0.139
BMI (kg/m^2^)	24.93 ± 4.60	24.95 ± 4.64	24.69 ± 3.69	24.37 ± 3.74	0.268
Smoking, n (%)	222(1.4)	150(1.0)	59(9.0)	13(28.3)	<0.001
Betel nut chewing, n (%)	63(0.4)	35(0.2)	24(3.7)	4(8.7)	<0.001

Next, we performed multivariate logistic regression analysis of the association of alcohol drinking with CKD in men and women (Tables 3 and 4). In both genders, the odds of having stage 3 CKD was positively associated with age, HTN, anemia, hyperuricemia, and proteinuria. High BMI (≥ 24 kg/m^2^) in men and smoking and hyperlipidemia in women were also associated with increased odds for stage 3 CKD. The inverse association of alcohol drinking and CKD was significant for men only. Further analysis of alcohol consumption indicated that the *p*-values for trend were significant for men and women.

**Table 3:** Multivariate logistic regression analysis of the association of alcohol consumption with chronic kidney disease in men based on three models^*^

**N = 11,900**	**Model 1**		**Model 2**		**Model 3**	
	**Multivariable OR**	***p***	**Multivariable OR**	***p***	**Multivariable OR**	***p***
	**(95% CI)**		**(95% CI)**		**(95% CI)**	
Age ≥ 65 years	5.53(4.97-6.15)	<0.001	5.54(4.97-6.17)	<0.001	5.07(4.52-5.70)	<0.001
Smoking			0.95(0.83-1.09)	0.460	1.02(0.89-1.18)	0.766
Betel nut chewing			1.09(0.91-1.32)	0.347	1.12(0.92-1.36)	0.270
Hypertension					1.63(1.46-1.83)	<0.001
Diabetes					1.05(0.90-1.21)	0.549
Anemia					2.14(1.78-2.57)	<0.001
Hyperlipidemia					1.15(0.89-1.50)	0.293
BMI 18.5-24/ BMI < 18.5					1.39(0.96-2.01)	0.083
BMI ≥ 24/ BMI <18.5					1.55(1.07-2.24)	0.021
Hyperuricemia					3.19(2.86-3.56)	<0.001
Proteinuria					2.27(1.96-2.62)	<0.001
Occasional drinking/Non-drinking	0.73(0.64-0.83)	<0.001	0.72(0.63-0.83)	<0.001	0.68(0.59-0.78)	<0.001
Frequent drinking/Non-drinking	0.61(0.46-0.81)	0.001	0.61(0.46-0.81)	0.001	0.47(0.35-0.63)	<0.001
	*P* for trend	<0.001	*P* for trend	<0.001	*P* for trend	<0.001
Non-drinking/Occasional drinking	1.37(1.21-1.56)	<0.001	1.38(1.21-1.58)	<0.001	1.48(1.29-1.70)	<0.001
Frequent drinking/Occasional drinking	0.84(0.63-1.13)	0.242	0.84(0.62-1.12)	0.236	0.69(0.51-0.94)	0.020
	*P* for trend	<0.001	*P* for trend	<0.001	*P* for trend	<0.001

*In this table and Table 4: Model 1 was adjusted for age alone; Model 2 was adjusted for age plus smoking and betel nut chewing; Model 3 was adjusted for all variables in Model 2 plus hypertension, diabetes, anemia, hyperlipidemia, body mass index, hyperuricemia, and proteinuria.

**Table 4:** Multivariate logistic regression analysis of the association of alcohol drinking with chronic kidney disease in women based on three models

**N = 15,353**	**Model 1**		**Model 2**		**Model 3**	
	**Multivariable OR**	***p***	**Multivariable OR**	***p***	**Multivariable OR**	***p***
	**(95% CI)**		**(95% CI)**		**(95% CI)**	
Age ≥ 65 years	8.88(8.01-9.85)	<0.001	8.90(8.02-9.87)	<0.001	6.65(5.95-7.43)	<0.001
Smoking			1.46(0.96-2.23)	0.081	1.62(1.05-2.50)	0.031
Betel nut chewing			1.19(0.58-2.45)	0.637	1.05(0.50-2.21)	0.908
Hypertension					1.45(1.30-1.62)	<0.001
Diabetes					1.11(0.96-1.28)	0.149
Anemia					1.81(1.56-2.11)	<0.001
Hyperlipidemia					1.36(1.08-1.71)	0.010
BMI 18.5-24/ BMI <18.5					0.81(0.58-1.12)	0.193
BMI ≥24/BMI <18.5					0.82(0.59-1.13)	0.214
Hyperuricemia					3.78(3.35-4.27)	<0.001
Proteinuria					2.15(1.85-2.49)	<0.001
Occasional drinking/Non-drinking	1.11(0.83-1.49)	0.474	1.07(0.80-1.43)	0.661	1.05(0.78-1.43)	0.734
Frequent drinking/Non-drinking	0.78(0.23-2.66)	0.695	0.69(0.20-2.37)	0.559	0.64(0.19-2.23)	0.487
	*P* for trend	<0.001	*P* for trend	<0.001	*P* for trend	<0.001

**Figure 2:**
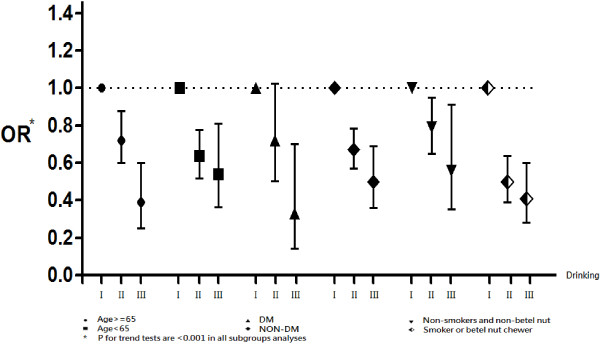
**Analysis of CKD risk in men who were non-drinkers (I, as the reference group), occasional drinkers (II), and frequent drinkers (III) with respect to age, presence of diabetes, and tobacco and betel nut usage.** The analysis was adjusted for age, smoking, betel nut chewing, hypertension, diabetes, anemia, hyperlipidemia, body mass index, hyperuricemia, and proteinuria.

**Figure 3:**
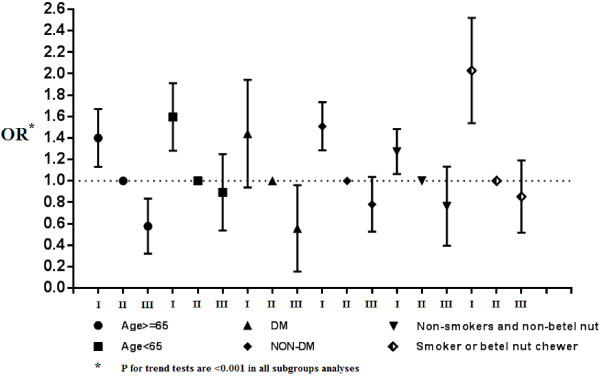
**Sensitivity tests by using occasional drinkers (II) as the reference group.** The adjustment variables were identical with the analysis in Figure 2.

Finally, we further analyzed the data for men who were self-classified as non-drinkers, occasional drinkers, and frequent drinkers (Figure 2) with adjustment of age, smoking, betel nut chewing, hypertension, diabetes, anemia, hyperlipidemia, body mass index, hyperuricemia, and proteinuria. This sub-group analysis indicated a significantly inverse association of drinking and CKD in three groups of men: those younger than 65 years and 65 years and older; those with and without DM; and those who did not smoke and did not chew BN and those who smoked or chewed BN. Further sensitivity tests by treating occasional drinker as the reference group revealed similar relationships among alcohol consumption and CKD (Table 3 and Figure 3).

## Discussion

Alcohol drinking is an important part of the lives of many people, and is related to the culture, religion, anthropology, and economic development of diverse populations throughout the world. Thus, it is important to assess the effect of alcohol consumption on human health. The present results indicate that alcohol drinking was inversely and significantly associated with stage 3 CKD in Taiwanese men after controlling for potential confounding covariates. In addition, trend analysis indicated that alcohol consumption seemed to be associated with lower odds of CKD in both men and women; also in men regardless of age, diabetes status, tobacco smoking, and BN chewing. These results imply a dose–response relationship of alcohol drinking and reduced odds of having stage 3 CKD.

Our finding of an inverse association between alcohol drinking and CKD is consistent with the findings of previous studies, although these other studies have used different criteria for classification of renal disease. For example, Savdie et al. reported that women and men who consumed three or more drinks daily had lower SCr than those who had two or fewer drinks daily [14]. Schaeffner et al. performed a prospective study of healthy men and reported a significantly lower risk of renal dysfunction (defined as SCr ≥ 1.5 mg/dL and eGFR ≤ 55 mL/min) in those who consumed at least 7 drinks weekly [16]. Reynolds et al. performed a prospective cohort study in China and reported a significant inverse relationship between alcohol consumption and risk of ESRD in men, and a stronger relationship in those who had more than 21 drinks weekly [17]. White et al. reported significantly reduced risk of CKD (defined as eGFR < 60 mL/min/1.73 m^2^) in Australian men who consumed at least 30 grams of alcohol daily [13]. Yamagata et al. reported ethanol consumption less than 20 g daily reduced the risk of developing renal dysfunction (defined as CKD stage 1 to 5) in both genders, and that this favorable effect was diminished with ethanol intake of more than 20 g daily [24]. However, there are also some contrary results. For example, consumption of more than two drinks daily was associated with increased risk of ESRD in an American population [11] and drinking 4 or more servings daily, especially when combined with smoking, was independently associated with increased CKD risk (defined as eGFR ≤ 60 mL/min/1.73 m^2^) in a different American population [12].

There are several potential explanations for the inverse association of alcohol consumption and CKD. The polyphenols in many alcoholic beverages are widely believed to have beneficial health effects due to their anti-oxidant properties. For example, long-term exposure to polyphenol-rich red wine led to enhancement of antioxidant defenses in rat plasma and kidney [25]. Long-term alcohol consumption may reduce kidney injury by induction of catalase [26], superoxide dismutase [27], or glutathione peroxidase [25]. Other animal studies have documented that alcohol can protect from development of renal ischemia/reperfusion injury [28,29], reduce renal arteriolar hyalinization independently of its effect on other cardiovascular disease risk factors [30], and prevent leukocyte recruitment and endothelial barrier damage [31].

Alcohol consumption also affects metabolism, and previous clinical studies indicated that moderate consumption is associated with increased high density lipoprotein (HDL) and plasma concentration of endogenous tissue-type plasminogen activator [32,33], thereby protecting against atherosclerosis. Moderate alcohol consumption also prevents development of atherosclerosis in subjects with type 2 DM [34]. Our data indicate that the inverse association of alcohol drinking with CKD appears to be independent of its effect on metabolism.

There are several limitations to this study. First, this study had a cross-sectional design, so we cannot definitively establish causality. Second, alcohol consumption data was self-reported according to a fixed questionnaire lacking in quantitative measurement, and is therefore susceptible to under-reporting. However, previous research has validated the use of self-reported alcohol drinking data [35,36] and this is considered acceptable in epidemiological research. Some research of heavy drinkers indicated under-reporting of consumption [37]. However, our results of trend testing indicated a significant association of amount of alcohol drinking and CKD, and this association would be stronger if there was under-reporting by heavy drinkers. A major strength of our study is that we used a large number of cases, allowing subgroup analysis. Second, we used gender-stratified analysis to document the different effects of alcohol drinking on CKD in men and women. In addition, previous research indicated that anemia, hyperuricemia, and BN chewing were associated with CKD [20,38-40], so we adjusted for these confounders in our multivariable analysis. Besides, we conducted a series of sensitivity tests to remove impacts of reverse causality. Further, our research is the first to focus on the association of alcohol consumption with patients who have stage 3 CKD, a critical yet easily overlooked group of patients. Further research should employ a longitudinal cohort design and should consider detailed alcohol drinking history (type of alcohol, amount of consumption, duration of consumption, etc.) to further establish causal relationships of alcohol consumption and CKD in both genders.

Based on our finding of an inverse association of alcohol consumption with CKD and the presence of many previous studies that documented the beneficial effects of moderate drinking on cardiovascular health, it is reasonable to cautiously recommend moderate alcohol drinking. The recent Canadian guidelines recommend no more than 14 and 9 drinks weekly for men and women, respectively [41]. However, alcohol also has an adverse effect on traffic safety, and can increase violent behavior, liver dysfunction, and some cancers, so the results of our study should be interpreted carefully.

## Conclusions

The prevalence of stage 3 CKD in drinkers was lower than non-drinkers (*p <* 0.001) and the percentage of drinkers with stage 3 CKD was less than that of non-drinkers. Multivariable analysis indicated that alcohol consumption was associated with a lower odd for stage 3 CKD in men only. Advanced age, hypertension, anemia, BMI of at least 24, hyperuricemia, and proteinuria were also associated with stage 3 CKD in men. Trend tests indicated lower odds of stage 3 CKD with increased alcohol consumption in both genders. Subgroup analysis indicated that alcohol consumption was also inversely associated with CKD in men regardless of age, diabetes status, and other risky behaviors. However, since alcohol has a well-known potential for abuse, the results in the current research should be interpreted cautiously.

## Abbreviations

CKD: Chronic kidney disease; ESRD: End-stage renal disease; EGFR: Estimated glomerular filtration rate; SCr: Serum creatinine; BN: Betel nut; DM: Diabetes mellitus; HTN: Hypertension; SBP: Systolic blood pressure; DBP: Diastolic blood pressure; TC: Total cholesterol; TG: Triglyceride; UA: Uric acid; FBG: Fasting blood glucose; ALT: Alanine aminotransferase; Hb: Hemoglobin; WBC: White blood cell; BMI: Body mass index; MDRD: Modification of diet in renal disease; OR: Odds ratio; CIs: Confidence intervals; HDL: High density lipoprotein.

## Competing interests

The authors declare that they have no competing interests.

## Authors’ contributions

YHH designed the study and drafted the manuscript. WHL provided administrative support and participated in the study design. HCP contributed to the interpretation of data and providing comments on the draft. YMC provided comments on draft and helped drafting the manuscript. CCH guided the statistical works and revised the manuscripts. All authors read and approved the final manuscript.

## Pre-publication history

The pre-publication history for this paper can be accessed here:

http://www.biomedcentral.com/1471-2369/14/254/prepub

## References

[B1] Coresh J Selvin E Stevens LA Manzi J Kusek JW Eggers P Van Lente F Levey AS Prevalence of chronic kidney disease in the United States JAMA 2007 298 2038 2047 10.1001/jama.298.17.2038 17986697

[B2] Chadban SJ Briganti EM Kerr PG Dunstan DW Welborn TA Zimmet PZ Atkins RC Prevalence of kidney damage in Australian adults: the AusDiab kidney study J Am Soc Nephrol 2003 14 S131 S138 10.1097/01.ASN.0000070152.11927.4A 12819318

[B3] Hsu CC Hwang SJ Wen CP Chang HY Chen T Shiu RS Horng SS Chang YK Yang WC High prevalence and low awareness of CKD in Taiwan: a study on the relationship between serum creatinine and awareness from a nationally representative survey Am J Kidney Dis 2006 48 727 738 10.1053/j.ajkd.2006.07.018 17059992

[B4] United States Renal Data System (USRDS) 2012 Annual Data Report[http://www.usrds.org/2012/pdf/v2_ch12_12.pdf]

[B5] Wu MJ Shu KH Liu PH Chiang PH Cheng CH Chen CH Yu DM Chuang YW High risk of renal failure in stage 3B chronic kidney disease is under-recognized in standard medical screening J Chin Med Assoc 2010 73 515 522 10.1016/S1726-4901(10)70113-1 21051028

[B6] Tanaka K Hara S Kushiyama A Ubara Y Yoshida Y Mizuiri S Aikawa A Kawatzu S Risk of macrovascular disease stratified by stage of chronic kidney disease in type 2 diabetic patients: critical level of the estimated glomerular filtration rate and the significance of hyperuricemia Clin Exp Nephrol 2011 15 391 397 10.1007/s10157-011-0420-6 21331740

[B7] Ito S Naritomi H Ogihara T Shimada K Shimamoto K Tanaka H Yoshiike N Impact of serum uric acid on renal function and cardiovascular events in hypertensive patients treated with losartan Hypertens Res 2012 35 867 873 10.1038/hr.2012.59 22573200 PMC3419971

[B8] Keith DS Nichols GA Gullion CM Brown JB Smith DH Longitudinal follow-up and outcomes among a population with chronic kidney disease in a large managed care organization Arch Intern Med 2004 164 659 663 10.1001/archinte.164.6.659 15037495

[B9] Go AS Chertow GM Fan D McCulloch CE Hsu CY Chronic kidney disease and the risks of death, cardiovascular events, and hospitalization N Engl J Med 2004 351 1296 1305 10.1056/NEJMoa041031 15385656

[B10] WHO Global status report on alcohol and health 2011 2011 Geneva: World Health Organization 37 41

[B11] Perneger TV Whelton PK Puddey IB Klag MJ Risk of end-stage renal disease associated with alcohol consumption Am J Epidemiol 1999 150 1275 1281 10.1093/oxfordjournals.aje.a009958 10604769

[B12] Shankar A Klein R Klein BE The association among smoking, heavy drinking, and chronic kidney disease Am J Epidemiol 2006 164 263 271 10.1093/aje/kwj173 16775042

[B13] White SL Polkinghorne KR Cass A Shaw JE Atkins RC Chadban SJ Alcohol consumption and 5-year onset of chronic kidney disease: the AusDiab study Nephrol Dial Transplant 2009 24 2464 2472 10.1093/ndt/gfp114 19307230

[B14] Savdie E Grosslight GM Adena MA Relation of alcohol and cigarette consumption to blood pressure and serum creatinine levels J Chronic Dis 1984 37 617 623 10.1016/0021-9681(84)90111-5 6746851

[B15] Cueto-Manzano AM Martinez-Ramirez HR Cortes-Sanabria L Management of chronic kidney disease: primary health-care setting, self-care and multidisciplinary approach Clin Nephrol 2010 74 99 104 10.5414/cnp74s099 20979973

[B16] Schaeffner ES Kurth T de Jong PE Glynn RJ Buring JE Gaziano JM Alcohol consumption and the risk of renal dysfunction in apparently healthy men Arch Intern Med 2005 165 1048 1053 10.1001/archinte.165.9.1048 15883245

[B17] Reynolds K Gu D Chen J Tang X Yau CL Yu L Chen CS Wu X Hamm LL He J Alcohol consumption and the risk of end-stage renal disease among Chinese men Kidney Int 2008 73 870 876 10.1038/sj.ki.5002774 18185503

[B18] Gupta PC Ray CS Epidemiology of betel quid usage Ann Acad Med Singapore 2004 33 31 36 15389304

[B19] Chen JW Shaw JH A study on betel quid chewing behavior among Kaohsiung residents aged 15 years and above J Oral Pathol Med: official publication of the International Association of Oral Pathologists and the American Academy of Oral Pathology 1996 25 140 143 10.1111/j.1600-0714.1996.tb00209.x 8860146

[B20] Hsu YH Liu WH Chen W Kuo YC Hsiao CY Hung PH Jong IC Chiang PC Hsu CC Association of betel nut chewing with chronic kidney disease: a retrospective 7-year study in Taiwan Nephrology (Carlton) 2011 16 751 757 10.1111/j.1440-1797.2011.01489.x 21736664

[B21] Liu WH Hsu CC Hsu YH Chewing areca nut as an independent risk factor for proteinuria in middle-aged men Kaohsiung J Med Sci 2013 29 214 220 10.1016/j.kjms.2012.08.037 23541267 PMC11916246

[B22] National Kidney Foundation K/DOQI clinical practice guidelines for chronic kidney disease: evaluation, classification, and stratification Am J Kidney Dis 2002 39 S1 S266 11904577

[B23] Wortmann RL Kelley WN Kelley WN, Harris ED, Ruddy S, Sledge CB Gout and hyperuricemia Textbook of Rheumatology 2005 7 Philadelphia: W.B. Saunders 1402 1448

[B24] Yamagata K Ishida K Sairenchi T Takahashi H Ohba S Shiigai T Narita M Koyama A Risk factors for chronic kidney disease in a community-based population: a 10-year follow-up study Kidney Int 2007 71 159 166 10.1038/sj.ki.5002017 17136030

[B25] Rodrigo R Rivera G Orellana M Araya J Bosco C Rat kidney antioxidant response to long-term exposure to flavonol rich red wine Life Sci 2002 71 2881 2895 10.1016/S0024-3205(02)02140-9 12377269

[B26] Orellana M Valdes E Fernandez J Rodrigo R Effects of chronic ethanol consumption on extramitochondrial fatty acid oxidation and ethanol metabolism by rat kidney Gen Pharmacol 1998 30 719 723 10.1016/S0306-3623(97)00342-X 9559324

[B27] Dreosti IE Manuel SJ Buckley RA Superoxide dismutase (EC 1.15.1.1), manganese and the effect of ethanol in adult and foetal rats Br J Nutr 1982 48 205 210 10.1079/BJN19820106 6889437

[B28] Giovannini L Migliori M Longoni BM Das DK Bertelli AA Panichi V Filippi C Bertelli A Resveratrol, a polyphenol found in wine, reduces ischemia reperfusion injury in rat kidneys J Cardiovasc Pharmacol 2001 37 262 270 10.1097/00005344-200103000-00004 11243416

[B29] Kahraman A Erkasap N Serteser M Koken T Protective effect of quercetin on renal ischemia/reperfusion injury in rats J Nephrol 2003 16 219 224 12768068

[B30] Burchfiel CM Tracy RE Chyou PH Strong JP Cardiovascular risk factors and hyalinization of renal arterioles at autopsy. The Honolulu heart program Arterioscler Thromb Vasc Biol 1997 17 760 768 10.1161/01.ATV.17.4.760 9108792

[B31] Shigematsu S Ishida S Hara M Takahashi N Yoshimatsu H Sakata T Korthuis RJ Resveratrol, a red wine constituent polyphenol, prevents superoxide-dependent inflammatory responses induced by ischemia/reperfusion, platelet-activating factor, or oxidants Free Radic Biol Med 2003 34 810 817 10.1016/S0891-5849(02)01430-2 12654468

[B32] Gaziano JM Buring JE Breslow JL Goldhaber SZ Rosner B VanDenburgh M Willett W Hennekens CH Moderate alcohol intake, increased levels of high-density lipoprotein and its subfractions, and decreased risk of myocardial infarction N Engl J Med 1993 329 1829 1834 10.1056/NEJM199312163292501 8247033

[B33] Ridker PM Vaughan DE Stampfer MJ Glynn RJ Hennekens CH Association of moderate alcohol consumption and plasma concentration of endogenous tissue-type plasminogen activator JAMA 1994 272 929 933 10.1001/jama.1994.03520120039028 7794308

[B34] Wakabayashi I Kobaba-Wakabayashi R Masuda H Relation of drinking alcohol to atherosclerotic risk in type 2 diabetes Diabetes Care 2002 25 1223 1228 10.2337/diacare.25.7.1223 12087023

[B35] Northcote J Livingston M Accuracy of self-reported drinking: observational verification of ‘last occasion’ drink estimates of young adults Alcohol Alcohol 2011 46 709 713 10.1093/alcalc/agr138 21949190

[B36] Giovannucci E Colditz G Stampfer MJ Rimm EB Litin L Sampson L Willett WC The assessment of alcohol consumption by a simple self-administered questionnaire Am J Epidemiol 1991 133 810 817 2021148 10.1093/oxfordjournals.aje.a115960

[B37] Parekh RS Klag MJ Alcohol: role in the development of hypertension and end-stage renal disease Curr Opin Nephrol Hypertens 2001 10 385 390 10.1097/00041552-200105000-00014 11342802

[B38] Keane WF Zhang Z Lyle PA Cooper ME de Zeeuw D Grunfeld JP Lash JP McGill JB Mitch WE Remuzzi G Risk scores for predicting outcomes in patients with type 2 diabetes and nephropathy: the RENAAL study Clin J Am Soc Nephrol 2006 1 761 767 10.2215/CJN.01381005 17699284

[B39] Mohanram A Zhang Z Shahinfar S Keane WF Brenner BM Toto RD Anemia and end-stage renal disease in patients with type 2 diabetes and nephropathy Kidney Int 2004 66 1131 1138 10.1111/j.1523-1755.2004.00863.x 15327408

[B40] See LC Kuo CF Chuang FH Shen YM Ko YS Chen YM Yu KH Hyperuricemia and metabolic syndrome: associations with chronic kidney disease Clinical rheumatology 2011 30 323 330 10.1007/s10067-010-1461-z 20411291

[B41] Hackam DG Khan NA Hemmelgarn BR Rabkin SW Touyz RM Campbell NR Padwal R Campbell TS Lindsay MP Hill MD The 2010 Canadian hypertension education program recommendations for the management of hypertension: part 2 - therapy Can J Cardiol 2010 26 249 258 10.1016/S0828-282X(10)70379-2 20485689 PMC2886555

